# Contactless Battery Sensing: A Survey

**DOI:** 10.3390/s26041365

**Published:** 2026-02-21

**Authors:** Saravana Ram Srinivasan, Pedro Callado de Paiva, Aditi Dharmadhikari, Lyall Sathishkumar, Christian Nwobu, Ningyue Mao, Guilherme Hollweg, Xuan Zhou, Xiao Zhang

**Affiliations:** College of Engineering and Computer Science, University of Michigan-Dearborn, Dearborn, MI 48128, USA; saravna@umich.edu (S.R.S.); ppaiva@umich.edu (P.C.d.P.); aditivd@umich.edu (A.D.); lyalls@umich.edu (L.S.); cnwobu@umich.edu (C.N.); ningyuem@umich.edu (N.M.); hollweg@umich.edu (G.H.); xuanzhou@umich.edu (X.Z.)

**Keywords:** battery sensing, wireless diagnostics, state of charge, machine learning, multi-modal, second-life battery

## Abstract

As demand for EVs (Electric Vehicles), WSNs (Wireless Sensor Networks), and IoT (Internet of Things) devices continues to grow, efficient battery health monitoring has emerged as a critical requirement. Conventional BMS (Battery Management System) designs rely on wired, centralized architectures, which are not only costly and less scalable but also highly prone to operational failures. To mitigate these inherent drawbacks, recent studies have shifted toward exploring wireless, low-power, and contactless alternatives. This paper reviews emerging sensing solutions and machine learning techniques for battery state and health estimation. It also examines WBMS (Wireless Battery Management System) advancements from theoretical frameworks to prototypes, covering health monitoring, cycle/discharge tracking, thermal management, and second-life reuse. Additionally, we discuss integrating techniques including EIS (electrochemical impedance spectroscopy), ultrasonic sensing with IoT systems and advanced machine learning models. Furthermore, it explores innovative diagnostic approaches and highlights algorithmic frameworks for real-time diagnostics. Overall, this work provides a comprehensive view of intelligent, wireless battery-monitoring technologies and identifies key challenges and research opportunities for scalable deployment in cyber–physical systems.

## 1. Introduction

The rapid growth of electric vehicles (EVs), unmanned aerial vehicles (UAVs), and grid-scale energy storage has significantly increased global reliance on lithium-ion batteries (LIBs) [1]. In 2023, the EV market alone consumed over 450 GWh of LIBs, with projections exceeding 1.5 TWh by 2030. Similarly, the commercial drone market, expected to reach $54.6 billion by 2030 [2], relies heavily on high-performance batteries to extend flight times. However, battery degradation, manifested as capacity fade, increased internal resistance, and thermal instability, remains a critical challenge that directly impacts safety, efficiency, and operational costs, making robust and real-time battery state monitoring essential to mitigate these risks [3].

Conventional battery health monitoring relies on wired sensors integrated into battery management systems (BMS), which provide real-time measurements of parameters including voltage, current, temperature, and impedance. For example, coulomb counting tracks state of charge (SoC) by integrating current over time [4], while electrochemical impedance spectroscopy (EIS) analyzes frequency-domain responses to diagnose aging mechanisms. Despite their widespread use, these methods face inherent limitations: scalability issues in large-scale deployments (e.g., grid storage with thousands of cells), added complexity from wiring harnesses [5], and limited sensitivity to early-stage degradation, particularly in sealed battery systems. Recent surveys have uncovered that many BMS-related failures stem from sensor or wiring faults [6], underscoring the need for more robust alternatives.

To overcome the limitations of traditional battery monitoring, researchers are developing contactless sensing techniques such as millimeter-wave radar, optical/thermal sensors, and near-field communication (NFC) [7,8]. These systems enable non-intrusive, physically isolated monitoring, enhancing safety by reducing electrical short circuits and thermal hazards while simplifying integration and maintenance [9,10]. When combined with edge Artificial Intelligence and signal processing algorithms, contactless sensing achieves robust real-time SoC and SoH (state of health) estimation, even under dynamic operating conditions [11]. However, challenges, such as environmental sensitivity, sensor alignment, and signal attenuation, require careful engineering optimization [12]. The emerging adoption of wireless signal analysis and remote sensing technologies facilitates next-generation monitoring systems that offer enhanced efficiency, scalability, and lower maintenance needs, which proves particularly essential for large-scale energy storage systems (ESS) and physically constrained applications [13].

Over the past decades, comprehensive reviews on battery-monitoring technologies have primarily focused on conventional BMSs, diagnostic algorithms, and data-driven condition assessment models [14]. These surveys, however, have largely overlooked wireless battery sensing technologies, especially their roles in real-time performance tracking, health assessment, and condition monitoring, and even individual studies on related topics suffer from narrow scopes. For example, Cao et al. (2024) examined wireless BMS architectures based on BLE, Zigbee, and NFC (addressing system integration challenges) but failed to explore battery signal acquisition methods or underlying sensing techniques [15]. Li et al. (2019) reviewed data-driven battery health prediction using machine learning, but their analysis was limited to wired sensing, neglecting wireless or embedded solutions for real-world scenarios [16]; Basic et al. (2023) developed an NFC-based wireless battery sensor for EVs and second-life battery (SLB) based applications (emphasizing secure data access and low-power operation) but constrained their study to single-interface implementations without comparing broader sensing approaches [17]; Mohammed (2021) acknowledged wireless monitoring’s potential for adaptive EV charging strategies but did not incorporate real-time sensing technologies into the analysis. These literature reveals a critical research gap: while wireless communication protocols in battery systems and data-driven battery health estimation [18] are well-covered, no comprehensive review links these two domains via wireless battery sensing and often overlook wireless capture/transmission of key physical signals (e.g., acoustic, electromagnetic, temperature, impedance) for continuous contactless battery monitoring [19].

To address this gap, this survey focuses on reviewing contactless sensing technologies in battery monitoring, organizing them into four integrated dimensions that combine classification and performance evaluation. **First**, we classify these technologies by the types of physical signals detected (e.g., temperature, acoustic, electromagnetic, impedance) and assess their accuracy and reliability for battery health monitoring, directly addressing the overlooked signal-capture gap mentioned earlier. **Second**, we analyze data processing methods (from local to cloud computing), focusing on their impact on real-time monitoring via latency and responsiveness, which bridges the disconnect between data-driven methods and wireless systems. **Third**, we examine the integration of machine learning and multimodal sensing techniques, as these enhance data fusion, fault diagnosis, and predictive accuracy, a key link between sensing data and health estimation. **Finally**, we assess their applicability across environments (EVs, ESS, SLB), considering adaptability to specific requirements, to reflect real-world deployment needs.

**Figure 1:**
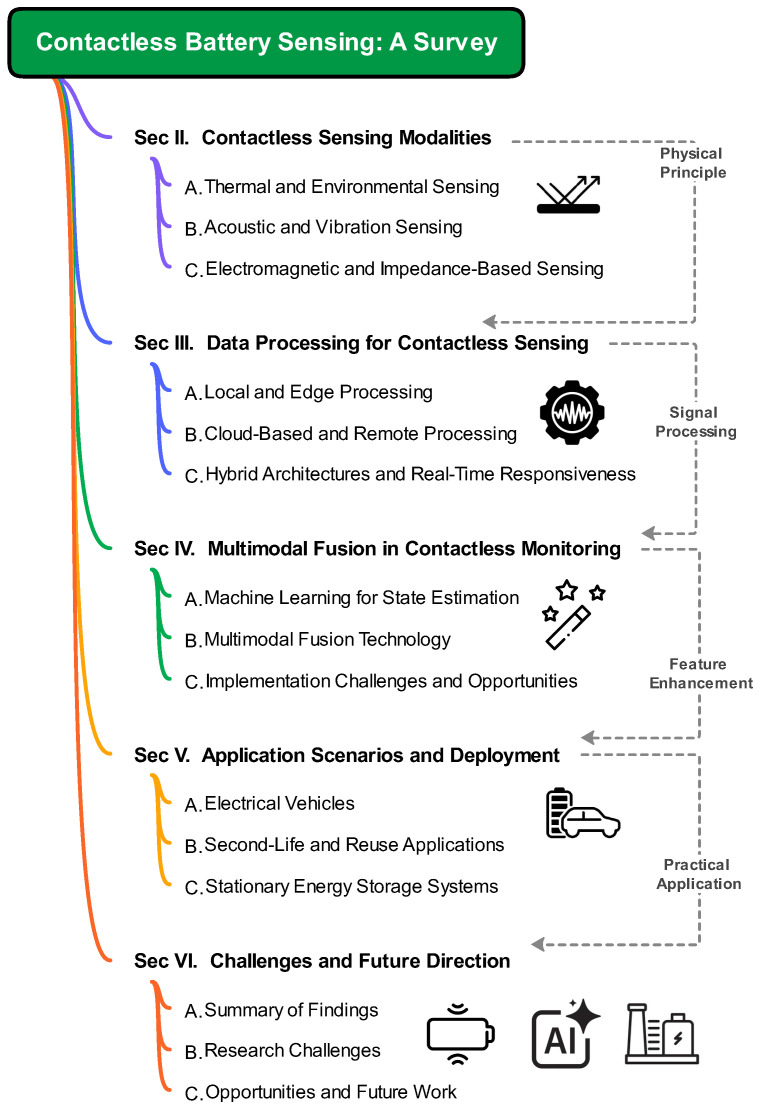
The survey structure and content coverage.

We aim to clarify the current research landscape, highlight effective approaches, further identify remaining gaps, and guide future work to improve the accuracy, scalability, and practical deployment of wireless battery-monitoring systems. The overview of this survey is illustrated in Figure 1. **Note**: This survey classifies a method as contactless sensing only if its sensing modality avoids direct electrical or mechanical contact with the battery cell. Systems that rely on contact-based sensors yet use wireless communication solely for data transmission are categorized as wireless monitoring, not contactless sensing. Accordingly, this review focuses on modalities that obtain battery states via non-contact physical interactions, independent of the transmission technology employed. As strictly defined herein, contactless sensing estimates battery states or safety metrics without any physical or electrical sensor attachment to cells or modules [20]. Representative examples include infrared thermography, acoustic/ultrasonic interrogation, electromagnetic field perturbation, radar imaging, and contactless impedance coupling. By contrast, setups using wired-on sensors (e.g., voltage, current, or temperature probes) with wireless data uplinks are labeled wireless monitoring systems [21]. This consistent distinction ensures conceptual rigor throughout the paper.

## 2. Contactless Sensing Modalities

**Figure 2:**
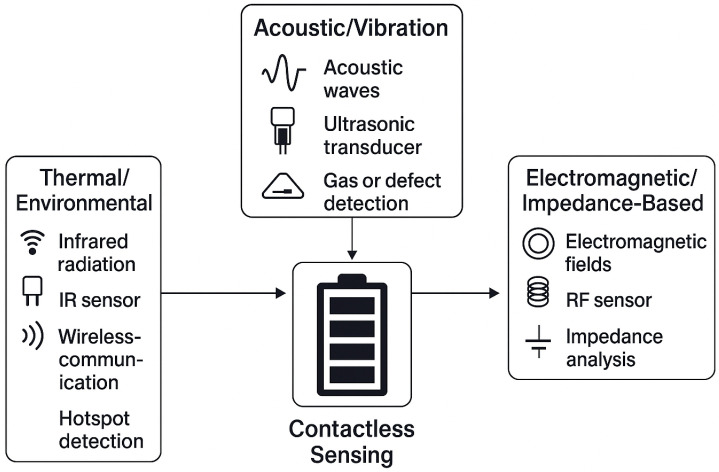
The existing contactless sensing modalities.

Contactless sensing is a key solution for battery health monitoring [15], solving core limitations of traditional contact-based methods (e.g., wiring complexity, invasiveness, limited access to dense or enclosed packs). These sensing modalities enable real-time, non-invasive detection of battery states (e.g., temperature, state of charge) and early faults (e.g., thermal runaway) by using physical signals including thermal estimation techniques [22], acoustic waves [23], and electromagnetic fields [24] that propagate without direct electrical or mechanical contact. We elaborate on three core contactless sensing categories (thermal/environmental, acoustic/vibration, electromagnetic/impedance-based) below (see Figure 2), focusing on their working principles, applications, performance metrics, and challenges.

### 2.1. Thermal and Environmental Sensing

Recent advances in BMS utilize infrared (IR) and non-contact temperature sensors for early thermal runaway detection in battery packs [25]. IR temperature sensors (e.g., pyroelectric sensor arrays [26]) directly measure surface temperature via emitted infrared radiation, enabling fast, non-invasive identification of overheating cells or hot spots in dense or inaccessible modules. Concurrently, wireless temperature-sensing solutions integrate two functionally distinct core components: non-contact temperature-sensing modules (e.g., IR detectors), a sensing modality that acquires temperature via non-contact signal detection and wireless data transmission components, which relay the measured data using communication technologies (e.g., passive UHF RFID tags [27] and NFC modules [8]). This setup supports flexible sensor placement in battery packs, even wire-inaccessible areas, with IR sensors capturing temperature data and UHF RFID/NFC enabling wireless data transfer [8,27].

While infrared and surface-based thermal sensing provide valuable early warning of abnormal heating, a key limitation is that they primarily capture surface temperature rather than the internal core temperature [28] of the cell. For battery management systems, core temperature is more directly related to electrochemical reaction rates, electrolyte stability, and thermal runaway risk [29]. As a result, contactless thermal sensing is most effective when combined with thermal modeling or data-driven estimation methods that infer core temperature from surface measurements [30] and operating conditions. This highlights an important research direction: integrating contactless thermal sensing with physics-based or machine-learning models to bridge the gap between observable surface signals and safety-critical internal states.

The effectiveness of such temperature-sensing solutions relies on two key metrics: high sensitivity and spatial resolution, both critical for effective surface-level temperature monitoring [31]. Detecting subtle temperature changes matters, just like precisely locating them, especially in dense cell packs where low-resolution sensing misses localized heating or faults [22]. Recent studies stress that monitoring accuracy also depends heavily on sensor count and placement in the pack [32]. Thus, distributed high-spatial-resolution sensor arrays enable accurate, real-time mapping of thermal events across cells, ensuring early detection of potential safety risks. In practical battery systems, however, temperature monitoring is typically achieved using a limited number of surface-mounted sensors, such as thermistors or thermocouples, since placing sensors on every cell surface is infeasible due to cost and system complexity considerations [33]. While infrared sensing enables fast and non-invasive surface temperature measurement, surface temperature does not directly represent the internal core temperature of a battery cell, providing only partial observability of the battery’s thermal state. Moreover, surface-mounted sensors are inherently unable to track rapidly varying internal core temperatures due to thermal diffusion delays between the cell core and the surface, an effect that becomes more pronounced under high current operation or transient conditions [22]. Yet, core temperature plays a critical role in electrolyte behavior, aging mechanisms, and thermal runaway initiation, making it a key variable for battery management systems. In practice, surface temperature measurements are coupled with model-based thermal estimation techniques that infer internal temperature. The accuracy of these estimations can be further improved by combining surface thermal data with complementary contactless signals, such as impedance-based, which correlate with internal electrochemical activity [33]. As a result, surface thermal sensing should be viewed as an enabling input for core temperature estimation rather than a direct substitute for internal measurements.

From a broader perspective, thermal and environmental sensing technologies offer distinct advantages for real-world battery packs and enclosures [10]. Their flexible installation and wireless data transmission support multi-sensor deployment in crowded or hard-to-reach areas without adding wiring complexity, boosting scalability and coverage [34]. They also enable non-invasive integration (no wiring or cell modification), further safeguarding battery integrity [35]. Conversely, their performance hinges on careful sensor placement and robust wireless communication, as dense enclosures, metal components, temperature, vibration, and electromagnetic interference can disrupt sensor operation and signal quality [9,12]. However, studies confirm these contactless solutions excel at enhancing battery safety and reliability when coverage and integration challenges are properly addressed.

### 2.2. Acoustic and Vibration Sensing

Acoustic emission (AE) and ultrasonic sensing techniques enable early-stage, non-invasive fault detection in electrochemical energy storage systems. In vanadium redox flow batteries (VRBs), pulse-echo methods monitor hydrogen evolution and estimate bubble flow rates [36]. In lithium-ion batteries, MCS sensors detect external short circuits and mechanical vibrations, with high temperature tolerance and deformation capacity. These sensors outperform traditional voltage-current monitoring, especially when integrated with Backscatter Communication (BackCom) for wireless, battery-free operation in sustainable, next-generation IoT systems.

The precise monitoring capabilities of such ultrasonic sensing technologies, in particular, stem from their sensitivity to signal changes inside batteries. Ultrasonic signals travel through battery electrolytes and structural components, revealing disruptions caused by gas formation or mechanical stress [37]. Variations in sound speed indicate changes in material properties, while increased attenuation highlights energy loss from scattering by bubbles or internal defects. These signals help capture critical failure modes such as hydrogen evolution, electrode loosening, or electrolyte delamination issues that are often undetectable by voltage or current measurements alone [38]. Thus, acoustic propagation offers deep insights into both the electrochemical and structural health of batteries.

However, despite these advantages, acoustic sensing technologies still face key challenges when scaled for broader practical use. Acoustic sensing technologies face challenges in signal stability, noise isolation, and bubble detection reliability. In VRBs, attenuation coefficients are more robust than sound speed for tracking SoC and hydrogen generation [36]. Missed detection may occur due to small or adsorbed bubbles, requiring precise sensor integration and configuration. BackCom offers low-power wireless data transmission but depends on stable ambient signals. Future acoustic telemetry systems may combine embedded AI and on-board processors to enhance real-time, autonomous battery health monitoring capabilities [39].

### 2.3. Electromagnetic and Impedance-Based Sensing

In recent advancements in contactless battery health monitoring, electromagnetic techniques, particularly radio frequency (RF) sensing [40] and electrochemical impedance spectroscopy (EIS) proxies, have gained momentum. These methods operate by detecting subtle perturbations in the electromagnetic field caused by internal changes in battery chemistry or structure [41]. For example, RF backscatter and resonant frequency shifts provide insights into electrolyte degradation and thermal effects [42]. Unlike classical EIS, which traditionally requires direct electrical contact with the target, contactless EIS proxies [43] infer impedance characteristics indirectly through electromagnetic coupling mechanisms such as coupling loops or non-invasive probes: rather than establishing a physical electrical connection to the measured object, they utilize electromagnetic fields generated by the probe or loop to interact with the target, thereby detecting changes in impedance without direct contact [44]. This modification bridges the gap between conventional EIS’s high accuracy and the demand for contactless sensing in scenarios where direct electrical contact is impractical or disruptive, while also reducing the need for invasive wiring and enabling seamless integration into compact and complex battery enclosures (e.g., EV battery packs) [45]. The challenge, however, lies in tuning the sensing frequency to balance penetration depth with sensitivity, especially in dense multi-cell configurations.

From a physical standpoint, contactless electromagnetic sensing techniques can be categorized based on their coupling mechanism and operating frequency. Inductive coupling relies on time-varying magnetic fields and operates in the near-field regime, typically at low frequencies ranging from a few kilohertz to several hundred kilohertz, where there is sufficient magnetic field penetration into conductive battery components [24,46,47]. Conversely, capacitive coupling also operates in the near-field regime but at higher frequencies, often in the megahertz range, to enhance sensitivity to small capacitance variations through electric field interactions [48]. Near-field RF techniques (e.g., NFC and backscatter) operate at even higher frequencies, providing increased sensitivity at the expense of reduced penetration depth and greater susceptibility to enclosure geometry and electromagnetic interference [49,50].

Within electromagnetic sensing technologies, near-field techniques and probe-based coupling form a practical subset, with unique advantages for specific battery-monitoring scenarios. A subset of electromagnetic sensing relies on near-field techniques and probe-based coupling [24]. This approach involves inductive or capacitive coupling to measure voltage and current without direct electrical contact. Contactless Rogowski coils and Hall-effect sensor arrays, for instance, have been demonstrated for real-time current monitoring while maintaining electrical isolation [51]. In multi-layer battery environments, these sensors can be placed near bus bars or conductor paths to retrieve dynamic current profiles. Additionally, integrating near-field signal acquisition with localized data processing (such as FPGA or edge-based microcontrollers) improves the system’s response time [52]. This consistency challenge under varying stresses directly points to a core requirement for all electromagnetic sensing technologies: robustness, which is critical for large-scale industrial use.

Robustness is a decisive factor for the widespread deployment of electromagnetic and impedance-based sensing [19]. Batteries deployed in different environments, ranging from consumer electronics to aerospace, exhibit varied chemical profiles and degradation mechanisms. Consequently, sensor systems must be resilient to EMI, temperature drift, and humidity [53]. Some research proposes adaptive calibration protocols and machine learning-based correction algorithms to tackle these inconsistencies [54]. Comparative studies have shown that impedance proxies retain relatively high accuracy across lithium-ion, lithium-iron-phosphate, and nickel-metal hydride chemistries when properly trained [55]. Nonetheless, reproducibility in field deployments continues to limit industrial uptake, prompting the need for hybrid approaches that combine electromagnetic sensing with auxiliary data streams such as thermal and acoustic signatures.

## 3. Data Processing for Contactless Sensing

**Figure 3:**
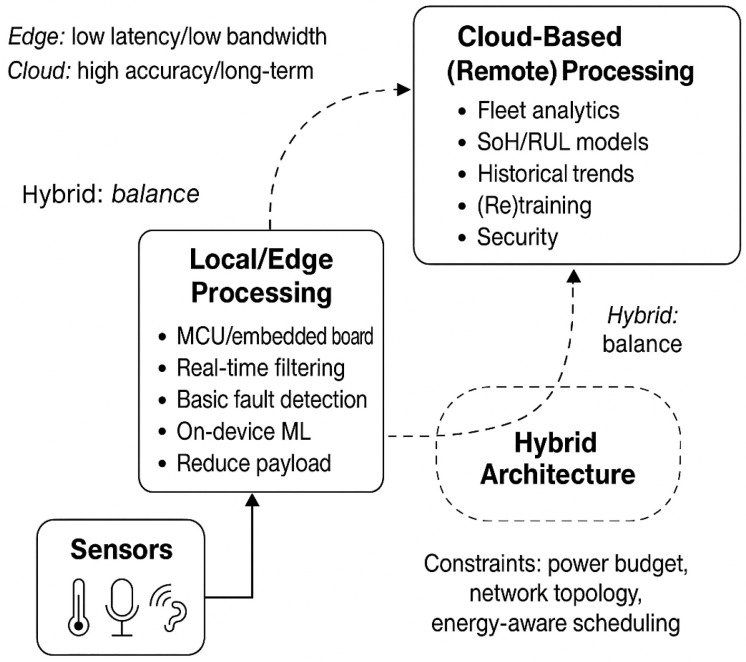
Three data processing modes and their relations.

Data processing is the core of reliable contactless battery sensing, determining the system’s responsiveness, scalability and adaptability. To meet diverse demands-from real-time monitoring to long-term health prediction-three interrelated processing paradigms have been developed. This section examines them systematically to form a comprehensive framework for optimizing contactless battery health monitoring, as shown in Figure 3.

### 3.1. Local and Edge Processing

Microcontroller-based signal processing has emerged as a foundational element in enabling contactless, embedded battery diagnostics, providing the hardware core for localized data handling. In [56], an STM32 microcontroller was used to extract radar features in real time and perform edge AI-based capacity classification while [6] demonstrated its application in distributed, ultra-low-power sensing with embedded ML for local signal analysis. Complementing this, [57] showed an ESP32 platform supporting real-time acquisition and preprocessing of battery parameters before wireless transmission, and [58] explored lightweight fault detection strategies (e.g., threshold-based alerts, statistical filtering) optimized for constrained hardware. Together, these implementations reduce system latency, improve energy efficiency, and lay the groundwork for continuous, external computation-free monitoring, highlighting localized intelligence as a cornerstone of scalable contactless battery health systems.

Building on this microcontroller-enabled embedded processing, edge computing further optimizes contactless monitoring by refining how localized computation interacts with data transmission. It reduces communication burden and power usage by shifting processing closer to the data source: ref [56] (consistent with its earlier microcontroller work) showed local signal processing/classification on an STM32 cutting data transmission volume, while [59]’s embedded smart sensing only sent critical insights over networks to lower energy costs and preserve bandwidth. Ref [60] extended this with an ESP32 that preprocessed signals and transmitted only essential health indicators, and [61] used on-device lightweight models for selective anomaly detection and data reporting. These advances confirm local processing not only ensures timely diagnostics but also boosts system efficiency-a key advantage that becomes even more critical in safety-critical scenarios like EVs, where the stakes of latency or inefficiency rise sharply.

Several studies address these demands: ref [62] introduced clustering algorithms to categorize faults and enhance reliability during dynamic driving; and [63] detailed real-time state estimation models tailored for automotive power batteries under variable loads. Supporting this, [64]’s rapid testing techniques enabled efficient SoH evaluations without sacrificing fault detection accuracy. Collectively, these designs build fault-tolerant EV systems where data-driven diagnostics safeguard passenger safety; embedded analytics and wireless alerts further strengthen onboard decision-making and response times. As EV adoption grows, this integrated tech stack offers a scalable path to safer battery integration across fleets and consumer platforms.

### 3.2. Cloud-Based and Remote Processing

While edge-based solutions excel at real-time, on-site data handling, they often face limitations in long-term trend analysis and large-scale fleet management–gaps that cloud-based processing frameworks are uniquely positioned to fill. As vital components for long-term battery health prediction, cloud systems deliver improved scalability, centralized analytics, and real-time decision support by harnessing advanced computational resources and historical datasets. Transmitting battery telemetry to the cloud enables more accurate estimations of state-of-health (SoH) and remaining useful life (RUL) [11]. For example, an ESP32-based architecture has been developed to stream data to the cloud for continuous monitoring and condition-based prediction [65], while IoT gateways combined with cloud-hosted machine learning have been employed to detect early-stage degradation in electric vehicle batteries [66]. This cloud-driven strategy is not limited to automotive applications: centralized data access has been leveraged for battery monitoring in aquatic systems [67], and cloud platforms have facilitated fleet-wide comparative analysis and adaptive trend modeling across large battery deployments [68]. Importantly, cloud frameworks alleviate onboard computational burdens and support periodic retraining of predictive models, thereby sustaining long-term estimation accuracy. With appropriate data encryption and access control mechanisms, these systems maintain high reliability while balancing operational performance and long-range visibility of battery assets [69].

Yet cloud reliance also exposes a key challenge for contactless battery sensing systems-especially those integrated with wireless and IoT communication: latency, bandwidth limitations, and data reliability issues. As noted in [56], delays in transmitting data to cloud servers can hinder timely decision-making in critical applications, a problem that ties directly to architecture choices for battery health monitoring. Centralized designs (e.g., [11,70]), which push all sensed data to a cloud or central unit, simplify long-term trend analysis but exacerbate latency and communication overhead-particularly for wide-ranging or remote systems. In contrast, distributed approaches (e.g., [71,72]) process signals locally at the sensing source, enabling faster anomaly responses and lighter network loads, but are constrained by embedded devices’ limited memory and computing power (restricting local execution of advanced algorithms). To reconcile these trade-offs, real-world deployments increasingly adopt hybrid architectures: they combine edge-level speed for real-time detection with cloud depth for long-term analytics, tailoring the balance to specific application requirements and system limitations.

### 3.3. Hybrid Architectures and Real-Time Responsiveness

Hybrid sensing architectures in battery systems employ local microcontrollers for fast, low-power preliminary detection such as identifying bubbles through changes in sound speed or measuring thermal irregularities while offloading intensive tasks like SoC and SoH prediction to cloud platforms [9]. This separation enables real-time responsiveness at the edge and leverages advanced machine learning models (e.g., LSTM-SVR) in the cloud [73]. Cloud services can integrate vast field data and high-resolution signals to model complex degradation behavior more accurately than standalone embedded systems. This division also reduces the computational burden on field devices, enabling lightweight designs and extending their operational lifetimes.

These hybrid systems achieve low-latency and reliable monitoring by combining local responsiveness with remote intelligence. Local MCUs quickly detect anomalies such as acoustic variability from gas bubbles to ensure fast alerts, while the cloud processes time-series trends using algorithms like recursive Gaussian Processes [74]. This dual-tier setup enables near-instant health checks while delivering high prediction accuracy ($R^{2}\approx 0.997$) for SoC and SoH [75]. The result is an architecture that balances speed and accuracy, supporting predictive maintenance and safety. Additionally, the use of spatiotemporal modeling techniques [76] ensures robustness even when edge data is noisy or incomplete.

However, deploying hybrid architectures in real battery systems presents challenges such as power constraints, data communication limits, and system complexity. Edge MCUs must use ultra-low-power components (e.g., 75nA SoC management units) and efficient RF modules to ensure minimal energy consumption [77]. Wireless communication introduces energy overhead and requires topology-aware planning (e.g., star or hybrid networks) [78]. Although cloud offloading reduces local computation, frequent transmission of high-resolution sensor data can strain limited battery resources, requiring intelligent energy-budget-aware scheduling [79]. System designers must carefully balance monitoring accuracy, data granularity, and energy efficiency to enable sustained real-time operation in field conditions.

## 4. Multimodal Fusion in Contactless Monitoring

**Figure 4:**
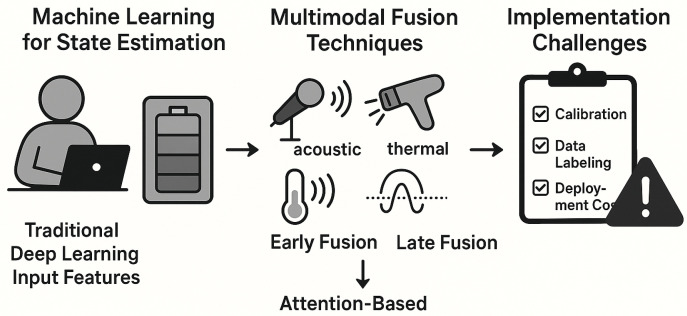
Multimodal fusion techniques for feature enhancement.

Building on the data processing for contactless sensing (which outlined workflows to extract insights from non-invasive sensor data), this section focuses on multimodal fusion to advance contactless battery health monitoring. While standalone contactless sensing and data processing enable basic state estimation (e.g., SoC, SoH), fusing diverse sensor data such as acoustic, thermal, and electromagnetic signals addresses single-signal limitations such as vulnerability to interference or incomplete capture of battery states. As shown in Figure 4, this section is organized into three parts: machine learning for state estimation using processed contactless data, key multimodal fusion techniques and architectures, and implementation challenges (and corresponding solutions) for scaling these systems.

### 4.1. Machine Learning for State Estimation

Traditional machine learning algorithms and models have been widely adopted for SoC and SoH estimation using non-invasive sensor data. Methods such as Support Vector Machine Classification (SVM), Support Vector Machine Regression (SVR) [80], K-means Clustering, Gaussian Processes [81], and linear and polynomial regressions are frequently employed to capture vital relationships in voltage, current, temperature, and impedance signals. Additionally, recent developments in deep learning models such as Long Short-Term Memory (LSTM) networks [82], autoencoders, and transfer learning models [83] have demonstrated enhanced accuracy in modeling complex time series and capturing non-linear battery degradation patterns. These models also adapt to diverse conditions and battery types, making them valuable for real-world applications.

The effectiveness of machine learning models for SoC and SoH estimation heavily relies on selecting specific input features extracted from sensor signals. Inputs such as thermal gradients, surface temperature [84], voltage and current curves, acoustic signatures [85], and impedance spectra [86] offer unique insights into battery behavior. For instance, thermal and expansion signals typically indicate localized heating or early cell degradation, while electrochemical impedance [87] and relaxation voltage [88] help capture subtle aging patterns. By mapping and properly selecting these diverse inputs to outputs like SoC or SoH, models can identify and predict degradation patterns that might otherwise remain undetected. This not only helps achieve high accuracy but also enables early fault detection in contactless battery health monitoring.

Although promising, machine learning approaches for contactless battery health monitoring face several implementation barriers [13]. To effectively deploy such systems, high-quality data is required, yet this can be difficult to obtain for diverse battery types and rare failure patterns. Techniques such as transfer learning and domain adaptation help address data scarcity [89], while proper feature selection and multi-modal sensor integration [90] remain critical for robust performance. Moreover, real-world battery packs may present issues with wireless interference, sensor placement, and environmental noise, which can affect data reliability and model accuracy if not suitably addressed. Additionally, while deep learning models excel in complex environments, their explainability is typically more limited compared to traditional methods. Hence, contactless battery health monitoring requires careful system design, training, calibration, and maintenance.

### 4.2. Multimodal Fusion Techniques

Recent studies present the fusion of acoustic [23], thermal [19], and electromagnetic signals as a key strategy to enhance the robustness of contactless battery health monitoring. By integrating data from expansion, surface temperature, and pyroelectric sensors [91], as well as impedance sensors and electromagnetic techniques (e.g., EIS, RFID, mmWave radar) [56], research demonstrates substantial gains in diagnostic accuracy and early fault detection. Unlike single-signal monitoring where faults like micro-short circuits or local overheating may go undetected, multimodal sensor fusion provides a more comprehensive assessment of batteries’ mechanical and thermal states. This approach directly addresses the limitations of individual sensors (e.g., thermal sensors’ susceptibility to environmental noise) and strengthens the overall reliability of battery monitoring in complex scenarios.

To efficiently integrate the aforementioned multimodal signals, three typical fusion architectures have been widely explored for battery monitoring: early fusion [92], late fusion [93], and attention-based mechanisms [94]. Early fusion combines signals (e.g., acoustic, thermal) at the feature input stage, allowing machine learning models to capture cross-signal correlations (e.g., the link between acoustic emissions and thermal runaway precursors) before prediction, studies show this significantly improves fault detection accuracy [95]. Late fusion, by contrast, merges outputs of separate single-signal models at the decision level; it offers strong robustness when individual signals are inconsistent or partially missing [96], such as when an impedance sensor fails temporarily. Advanced approaches, including autoencoders (for dimensionality reduction and noise filtering [97]) and deep learning models with attention mechanisms, further optimize performance by prioritizing high-value features (e.g., transient voltage fluctuations over steady-state signals), enabling more precise signal integration in practical diagnostics.

The combined strengths of these fusion architectures make multimodal techniques highly reliable for battery diagnostics under variable and challenging conditions. For instance, in electric vehicle (EV) battery packs where temperature swings, vibration, and wireless interference are common, fusing expansion, surface temperature, and impedance signals mitigates the impact of degraded or noisy sensors, maintaining overall output accuracy [96]. Beyond accuracy, these approaches also enable earlier fault detection warnings: for example, fusion of acoustic and thermal signals can identify electrode cracking 2–3 charge cycles earlier than single-modal monitoring. By reducing reliance on individual sensors and bridging complementary signals (e.g., using electromagnetic data to validate thermal anomalies), multimodal fusion ensures consistent, reliable operation even in adverse environments addressing a critical gap in real-world battery health management.

### 4.3. Implementation Challenges and Opportunities

The implementation of multimodal fusion-based contactless battery health monitoring systems presents several obstacles [35,98], such as sensor calibration, data labeling, and deployment costs, as previously discussed. Correct sensor calibration and placement are critical [22], as improper alignment or insufficient/wrong calibration can lead to inaccurate readings and reduced model reliability. Moreover, it is crucial to adequately track inputs and outputs to obtain accurate SoC and SoH estimations from models. However, the need for large, labeled datasets to train ML models poses a challenge [90]: collecting high-quality, labeled battery health data is often difficult, especially for rare failure cases. Lastly, deployment costs remain a barrier, as adding reliable sensors and wireless modules [99] while ensuring secure BMS integration increases system complexity and expenses. Research, however, shows ongoing advances toward compact, cost-effective sensor designs.

When it comes to large battery arrays and diverse applications, scalability remains a central challenge. While wireless and distributed sensor systems enable practical deployment across cells, ensuring reliable data collection and maintaining calibration grow increasingly complex as system size expands. Domain adaptation methods such as transfer learning [100,101] are essential for enabling models to generalize across different battery cells [102], pack configurations, operational environments, and applications. These methods leverage patterns and knowledge from previously trained models, helping mitigate the scarcity of labeled data [103]. Still, achieving robust scalability requires further advancements in sensor integration, data management, and model adaptation.

Looking forward, several promising research directions can address current limitations in contactless battery health monitoring. Self-supervised [104] and semi-supervised [105,106] learning reduce reliance on accurate labeled datasets by using unlabeled or partially labeled data. Federated [107] and distributed learning enable privacy-preserving training and cross-device knowledge sharing, supporting widespread deployment without centralized data. Additionally, advances in multi-modal fusion, adaptive algorithms, and edge computing will boost diagnostic accuracy, robustness, and real-time performance. As research progresses, these technologies should solve key challenges like scalability, data scarcity, and deployment complexity.

## 5. Application Scenarios and Deployment

Wireless and contactless battery-monitoring systems are critical across diverse real-world contexts, each with unique operational constraints, safety demands, and performance requirements. These contexts span EVs’ harsh dynamic environments, grid-scale ESS’ stationary long-duration needs, and second-life batteries’ uncertain reuse. This section examines how these technologies address scenario-specific challenges: for EVs, overcoming EMI, vibration, and space limits while integrating with automotive-grade BMS and safety standards; for second-life reuse, solving health uncertainty, enabling non-invasive maintenance, and scaling WSN deployment; and for stationary ESS, developing diagnostic tools for efficient sorting, triage, and long-term reliability. These analyses highlight contactless sensing’s adaptability and role in safer, more sustainable battery operation across key industry use cases.

### 5.1. Electric Vehicles

EV battery-health monitoring must operate amid aggressive electrical and mechanical stressors. High-voltage inverters and fast-switching power electronics create broadband interference that can corrupt wireless links; several teams note 2.4 GHz solutions are especially susceptible, motivating alternatives like NFC for short-range, interference-resilient sensor readouts and authenticated pack access [8]. Tight pack packaging and long wiring harnesses introduce galvanic isolation and connector challenges; moving to cell-level sensing (wired or wireless) reduces weakest-cell blind spots but tightens space for antennas, shielding, and thermal isolation. Real-world experiments inside EV packs highlight stringent QoS and deterministic sampling requirements; channel-hopping MACs (e.g., IEEE 802.15.4e/2015 TSCH) mitigate multi-path and external interferers via time-slotting and frequency diversity, improving reliability in noisy enclosures [6]. Mobility brings intermittent backhaul, as IoT/telematics paths (cellular/Wi-Fi) face connectivity gaps, latency, and packet loss, so edge processing, buffering, and store-and-forward become essential [4]. Finally, the thermally active pack demands sensing accurate across steep gradients; thermal management constraints limit redundancy and dictate careful placement and hardening of sensors against shock and vibration, with calibration strategies to suppress drift over a vehicle’s life [108].

The integration of wireless and contactless sensors into automotive battery monitoring offers a promising pathway to overcome EMI, vibration, and space-related challenges in EV packs. Millimeter-wave radar has demonstrated the ability to track lithium battery capacity with high accuracy, enabling real-time state estimation without invasive probes [56]. Similarly, piezoelectric and pyroelectric thin-film sensors provide continuous mapping of thermal and mechanical stress across cell surfaces, maintaining durability during dynamic drive cycles [91]. Wireless thermal monitoring platforms based on passive RFID tags achieve meter-scale readout distances with precise temperature tracking, making them suitable for harsh charging environments [77]. In addition, low-power wireless protocols such as IEEE 802.15.4e/2015 TSCH have been validated for in-pack communication, using frequency hopping and time-slotting to maintain robust links under EMI [6]. Automotive-focused wireless cell sensors further reduce wiring complexity, eliminate galvanic isolation issues, and enable per-cell SoC and SoH estimation [1]. While these advances confirm the viability of contactless sensing, their deployment in production EVs requires co-design with automotive qualification standards to ensure electromagnetic resilience, packaging robustness, and long-term fault tolerance.

Under the operating conditions typical of electric vehicle battery packs, wireless communication performance is strongly affected by electromagnetic interference, metallic enclosures, and tight packaging constraints. These factors lead to noticeable differences in how various wireless protocols behave in practice. Short-range technologies such as NFC are commonly used for in-pack diagnostics and configuration tasks because their near-field operation provides stable communication and predictable latency in high-interference environments [109]. Passive and semi-passive UHF RFID approaches are attractive for low-power temperature and health monitoring, as they reduce wiring complexity, but their effectiveness can be limited inside densely packed battery modules unless antenna placement and shielding are carefully considered. Bluetooth Low Energy supports flexible data exchange and higher throughput, although experience from automotive settings suggests that its reliability can degrade near high-voltage components due to packet loss and interference [110]. These observations indicate that wireless protocol selection in EV battery systems must be guided by pack-level constraints rather than nominal specifications, and that combining multiple communication approaches is often necessary to achieve an acceptable balance between reliability, power efficiency, and scalability [111].

The adoption of wireless and contactless sensing in automotive battery management systems introduces complex challenges related to functional reliability and regulatory compliance. Signal integrity and synchronization must be preserved despite the harsh electromagnetic environment of high-voltage inverters and converters, where noise can disrupt deterministic data exchange [6]. The asynchronous behavior of low-power wireless protocols further complicates fault detection, as milliseconds of delay can affect cutoff mechanisms for overcharge, short-circuit, or thermal runaway protection [8]. Ensuring compliance with functional safety standards such as ISO 26262 and electronic component qualifications like AEC-Q100 requires rigorous hardware validation, environmental stress testing, and long-term fault-tolerance strategies [1]. Integration also demands redundant sensing and failsafe firmware logic to keep critical protection features operable even under partial wireless failure [53]. Beyond reliability, interoperability remains a barrier: fragmented data formats and non-uniform communication frameworks hinder seamless deployment across heterogeneous EV platforms [112]. Addressing these issues requires a co-design philosophy where sensors, embedded controllers, and wireless protocols are jointly engineered for resilience, while standardization efforts converge on unified data exchange and qualification practices to ensure safety without sacrificing scalability.

### 5.2. Second-Life and Reuse Applications

**Figure 5:**
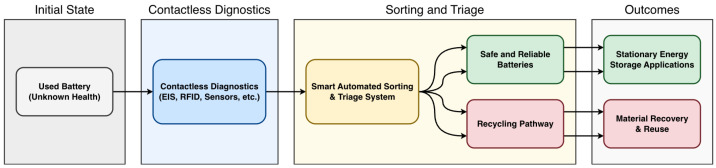
Workflow of contactless State of Health (SoH) diagnostics and triage for second-life battery management.

Grid-scale and industrial energy storage systems (ESS) [113], which range from lithium-ion packs to vanadium redox flow batteries (VFBs), require robust monitoring to ensure operational safety, longevity, and reliability across diverse usage scenarios. Factors such as extreme temperatures, frequent cycling, partial discharge events, and hydrogen gas generation in redox chemistries accelerate degradation and introduce operational safety concerns [114]. Second-life EV batteries reused in stationary applications often lack comprehensive monitoring infrastructure, driving demand for lightweight, non-invasive diagnostics and accurate SoH estimation methods. Effective monitoring directly supports grid resilience, decarbonization goals, and the long-duration storage required for high-penetration renewable energy integration across distributed energy networks, as illustrated in Figure 5.

Non-invasive sensing techniques, such as ultrasonic pulse-echo (for gas bubble detection), infrared thermography, and voltage-based machine learning, offer real-time battery health insights without disrupting normal operation or requiring physical teardown. In VFBs, pulse-echo systems reliably predict hydrogen evolution and assess the SoC of anolyte fluids, thereby preventing thermal or pressure-related failures during charging cycles [36]. In lithium-ion systems, acoustic resonance, sample entropy, or entropy-based methods can flag internal short circuits or over-discharge damage before they escalate [115]. These fast, modular diagnostics improve safety, reduce maintenance costs, and enable precise fault localization—a feature especially critical in modular or second-life battery applications deployed in remote locations.

Wireless Sensor Networks (WSNs), built on low-power microcontrollers (MCUs) and mesh protocols, enable scalable, cost-effective battery monitoring in both stationary and mobile ESS applications [116]. Whether deployed in second-life lithium packs, vanadium redox flow batteries, or hybrid microgrids, these networks support distributed sensing, rapid fault alerts, and cloud-based synchronization for historical trend analysis. The study in [12] discusses devices such as ESP32 development boards or Raspberry Pi gateways, which gather voltage, current, acoustic, and temperature data for low-latency real-time remote diagnostics. RESTCONF-based APIs, data compression techniques, and adaptive duty cycling protocols help maximize sensor lifetime and minimize bandwidth, making WSNs ideal for real-time, long-term monitoring in industrial, smart grid, and off-grid storage environments.

### 5.3. Stationary Energy Storage Systems

Second-life battery applications face a significant challenge of health uncertainty, as their true condition is usually unknown due to missing usage history and unclear degradation mechanisms. This is because second-life batteries often lack extensive measurement and monitoring infrastructure [117]. Without accurate SoH assessments [118], substantial discrepancies can arise between the actual and estimated health of batteries, compromising the safety and performance of repurposed cells. Furthermore, vital health indicators, such as residual capacity, internal resistance, and electrochemical component conditions are difficult to determine directly in second-life batteries, adding uncertainty to the reuse process and posing barriers to the safety and reliability of reused-battery applications.

Contactless diagnostic methods, such as EIS proxy, multimodal sensor fusion, RFID, and contactless capacity sensing, have gained momentum as tools for rapidly assessing the safety and usability of second-life batteries, enabling the estimation of their SoH and fault detection. Moreover, these technologies allow for the efficient assessment of large numbers of used batteries without the need for disassembly or invasive cell testing [119]. By providing accurate health information and supporting quick safety checks, contactless diagnostics assist in the selection and reuse of batteries, significantly reducing risks and improving the overall efficiency of second-life battery management.

The integration of contactless diagnostic techniques into smart, automated sorting and triage systems enables efficient and reliable management of second-life batteries. These systems ensure that only batteries meeting stringent safety and performance thresholds are repurposed, minimizing the risk of failure in second-life deployments. By rapidly assessing their SoH and safety status, these systems can direct batteries to the most suitable applications or recycling pathways based on their post-use condition [120]. Early-stage, non-invasive SoH assessment [121] facilitates classification and optimizes resource allocation, reducing human error and time-consuming manual labor. Lastly, such smart integration supports scalable and sustainable battery lifecycle management by minimizing waste and maximizing the useful life of each cell.

## 6. Challenges and Future Trend

### 6.1. Summary of Findings

This survey synthesizes recent progress in wireless and contactless battery-monitoring systems across four central dimensions. First, we examine the multiple physical signals leveraged for state estimation, including temperature (monitored via distributed arrays, surface, and pyroelectric sensors), acoustic signals, mechanical sensing data, electromagnetic methods (such as RFID/NFC), and impedance-based techniques. These physical signals are key for tracking battery health, detecting faults early, preventing thermal runaway, and monitoring aging processes. Second, we analyze data processing strategies ranging from feature extraction and signal fusion to real-time edge and cloud computation, and emphasize their impact on system latency, responsiveness, and accuracy. Third, we highlight the integration of traditional machine learning, deep learning, and multimodal sensing techniques; when combined, these techniques enhance predictive accuracy, enable advanced fault diagnosis, and support adaptive monitoring across diverse conditions. Finally, we assess the applicability of these technologies in real-world scenarios (including EVs, stationary storage, IoT deployments, and second-life battery use), while accounting for specific adaptability requirements and scalability limitations.

Advances in contactless battery monitoring have highlighted promising wireless and passive sensing technologies, such as UHF RFID, NFC modules, and pyroelectric sensor arrays, for scalable, non-invasive monitoring across systems of distinct sizes. Multimodal sensor fusion, which integrates temperature, acoustic, and impedance signals [122], has substantially improved diagnostic accuracy and early fault detection, while also demonstrating a transformative enhancement in SoC estimation across batteries under diverse operating conditions. Furthermore, developments in edge computing and compact sensor design have enhanced real-time analytics and reduced deployment barriers. Nevertheless, persistent challenges include sensor calibration and placement, the collection of high-quality labeled data, system cost, wireless interference, and ensuring model robustness across diverse applications.

Contactless battery-monitoring technologies offer significant value in terms of safety, sustainability, and operational efficiency. By enabling early detection of faults and thermal runaway risks through non-invasive, real-time sensing, these systems play a vital role in preventing hazardous incidents and maintaining battery integrity. Moreover, these approaches reduce wiring complexity and physical intrusion, thereby providing more sustainable battery management and facilitating the application of second-life batteries and large-scale energy storage. Additionally, advancements in compact design and scalable deployment enhance efficiency, lower installation costs, and support seamless integration in electric vehicles, stationary energy storage systems, and Internet of Things networks. Collectively, these benefits position contactless monitoring as a disruptive enabler for safer, more sustainable, and more efficient energy systems.

### 6.2. Research Challenges

**Figure 6:**
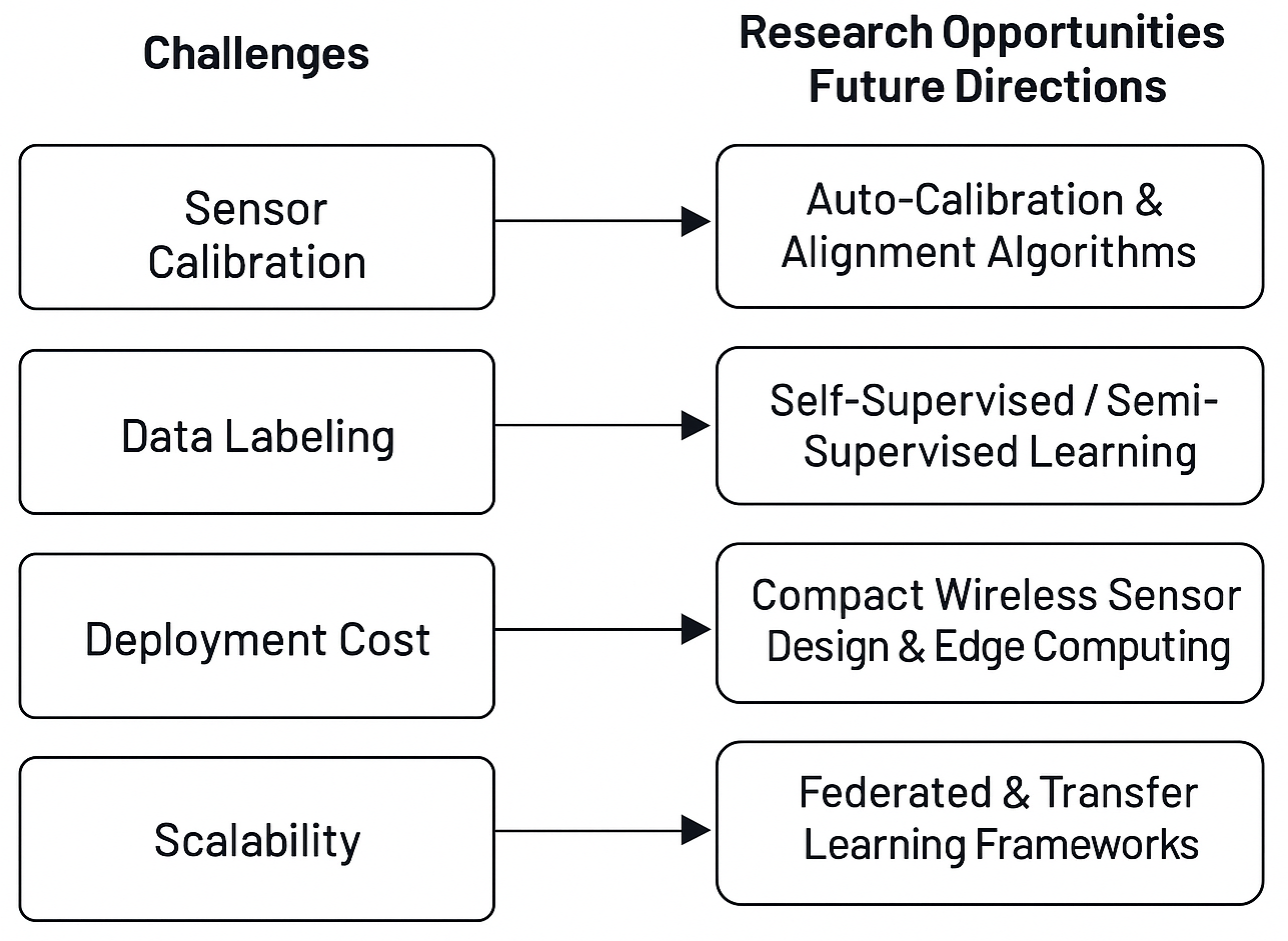
Challenges and future direction.

Despite notable advancements in wireless and contactless battery monitoring, several technical barriers persist that hinder scalability and real-time reliability as illustrated in Figure 6. Signal quality degradation is a persistent issue, often caused by electromagnetic interference, attenuation, or packet loss, factors that reduce the fidelity of impedance, temperature, or radar-based measurements. Hardware limitations further constrain performance: miniaturized low-cost sensors struggle with limited battery life, restricted sampling resolution, and thermal instability under harsh conditions. These trade-offs are magnified in embedded and vehicular environments, where strict power budgets and compact enclosures limit design flexibility. Data sparsity also remains a core challenge: insufficient failure data, sparse labeling, and short longitudinal records undermine the accuracy of supervised learning and heuristic models. Generalization across domains is still problematic, as transfer learning and adaptation methods remain sensitive to noise and fluctuating operating conditions. Interoperability adds another layer of difficulty, as non-standardized communication protocols and fragmented platforms hinder seamless integration. Addressing these issues requires low-noise sensor design, robust wireless links, and efficient edge analytics to ensure consistent operation. Ultimately, standardized frameworks and richer multimodal datasets are needed to develop trustworthy and scalable health monitoring solutions.

A closely related challenge is the limited observability of internal battery thermal states using non-invasive sensing alone. While contactless temperature sensing techniques provide valuable surface-level information, the accurate estimation of core temperature remains difficult, particularly in the presence of internal thermal gradients and dynamic operating conditions. Since core temperature directly influences electrolyte stability, aging behavior, and safety-critical failure mechanisms, improving its estimation from external measurements remains an open research problem. Addressing this challenge requires advances in thermal modeling, multimodal sensing integration, and adaptive estimation algorithms.

A second major challenge concerns the seamless integration of wireless and contactless health monitoring systems with existing battery management systems (BMS) under strict power constraints [123]. Conventional BMS platforms are designed around deterministic wired interfaces, while wireless modules introduce asynchronous behavior, latency, and packet collisions issues that complicate fault detection and closed-loop control. The limited power budget of sensor nodes further restricts sampling frequency, signal conditioning, and onboard analytics, particularly in passive or energy-harvested configurations [124]. These limitations are amplified when advanced functions such as edge inference, adaptive calibration, or secure communication are implemented, as they significantly increase duty cycles and reduce operating lifetime [125]. Co-locating wireless modules near high-current buses or DC/DC converters also generates voltage ripple and electromagnetic noise, which can impair stability unless carefully mitigated through isolation and filtering. Additionally, heterogeneous hardware designs and the lack of standardized power interfaces across BMS vendors create compatibility barriers that limit interoperability [126]. Addressing these challenges requires the coordinated co-design of energy-aware architectures that align sensor power profiles, communication schemes, and diagnostic thresholds with existing BMS logic. Such unified integration strategies are essential for balancing accuracy, safety, and longevity in next-generation battery management.

A third major barrier to the large-scale deployment of wireless battery-monitoring systems is the lack of standardized frameworks for sensor interfacing, communication protocols, and data representation. Existing solutions frequently rely on proprietary firmware, custom payload structures, and vendor-specific interfaces factors that restrict reuse across platforms and drive up integration costs. Variations in physical layers, such as Bluetooth Low Energy (BLE), Zigbee, NFC, and IEEE 802.15.4, further complicate interoperability, as differences in packet structures and command-response logic hinder the portability of sensor modules between manufacturers. The absence of harmonized formats for transmitting critical parameters, including State of Health (SoH), impedance, and thermal profiles creates additional challenges when integrating with cloud-based diagnostic platforms or over-the-air update systems. These inconsistencies delay validation and certification processes while limiting the scalability of predictive maintenance strategies and fleet analytics tools. To overcome these issues, collaborative efforts among original equipment manufacturers (OEMs), sensor suppliers, and standards bodies are essential to define unified data exchange formats, modular hardware interfaces, and interoperable middleware layers. Establishing such frameworks would enable plug-and-play deployment, reduce integration overhead, and accelerate the adoption of cross-platform monitoring solutions in next-generation electric vehicle ecosystems.

### 6.3. Opportunities and Future Work

Emerging advances in MEMS-based sensors, self-powered sensors and flexible electronic platforms are opening new opportunities for non-intrusive, real-time monitoring of battery health in constrained automotive environments [36]. These next-generation sensors can conform to irregular cell geometries while maintaining resilience to thermal stress, vibration, and electromagnetic interference, which ensures reliable signal acquisition [10]. Meanwhile, breakthroughs in machine learning, particularly transformer architectures and multimodal pretraining, enable the fusion of heterogeneous inputs such as impedance spectra, charging curves, and thermal profiles [127]. By leveraging these models, complex degradation pathways can be captured more effectively, even when labeled datasets are sparse or incomplete. This combination of adaptive sensing and data-driven intelligence has the potential to significantly improve the accuracy and robustness of SoH estimation. Looking ahead, such innovations pave the way for self-learning and adaptive monitoring frameworks that can dynamically adjust to evolving operating conditions in EVs.

Advancing wireless and AI-driven battery monitoring requires large-scale field trials to validate sensor reliability under diverse load profiles, thermal environments, and EMI-prone conditions. These trials should generate standardized, high-resolution datasets that capture multimodal signals, including voltage, current, impedance, and thermal responses, across different battery chemistries and degradation states. Such datasets are critical for reproducible research and provide the foundation for robust benchmarking of algorithms and sensor designs. Establishing open benchmarks with clearly defined evaluation protocols for SoH and SoC estimation would enable fair cross-model comparisons and accelerate innovation [74]. To be impactful, these benchmarks must reflect real-world variability and ensure the consistent reporting of data quality, sampling rates, and uncertainty measures. Collaborative initiatives between industry, academia, and standards organizations are vital to coordinate dataset creation, benchmarking, and governance. Collectively, these efforts will create a transparent pathway toward the scalable and certifiable deployment of next-generation battery-monitoring systems.

Contactless sensing offers transformative opportunities for future smart battery systems by providing real-time, non-invasive access to parameters such as temperature gradients, strain, and localized impedance. These modalities, including mmWave radar, piezoelectric films, and NFC-based readouts, enable early detection of safety-critical events like internal shorts or thermal runaway precursors. By supporting predictive maintenance, they can reduce downtime, extend operational life, and enhance reliability in EVs and grid-scale storage. Continuous sensing also feeds digital twin models, where virtual replicas forecast performance and degradation under dynamic operating conditions. In connected ecosystems, these data streams integrate seamlessly with cloud-based analytics and over-the-air optimization. Such integration enables adaptive charging strategies, fleet-wide diagnostics, and second-life battery assessments. Ultimately, standardized contactless platforms will become central to autonomous energy management, improving safety, sustainability, and scalability across smart infrastructure.

## 7. Conclusions

This paper reviews recent advances in wireless and contactless battery sensing for modern battery health monitoring. By investigating thermal, acoustic, electromagnetic, and impedance-based methods (see Table 1 and Table 2), we show that non-invasive sensing can effectively characterize internal battery behavior while reducing wiring complexity and safety hazards. We emphasize that hardware alone is insufficient: accurate SOC and SOH estimation increasingly depends on intelligent data processing and learning-based interpretation. Evaluations of edge, cloud, and hybrid architectures demonstrate the need to balance real-time responsiveness and long-term health analysis. In applications including electric vehicles, second-life batteries, and stationary energy storage, key deployment challenges include power constraints, wireless reliability, limited labeled data, and poor compatibility with conventional BMS. Multimodal data fusion is identified as critical to enhancing robustness and reducing uncertainty in contactless diagnostics. Future progress requires low-power sensor design, adaptive and self-supervised learning, and standardized datasets to enable practical, scalable implementation. Overall, contactless battery sensing represents a promising and essential direction toward safer, more reliable, and sustainable battery management in next-generation energy systems.

## Figures and Tables

**Table 1:** Summary of contactless sensing modalities in this review.

Sensing Modality	Physical Signal	Processing Mode	ML Integration	Key Advantages	Key Limitations
Thermal/IR	Surface temperature, heat radiation	Edge/Cloud	LSTM, regression models	Early thermal runaway detection, non-invasive	Surface-only sensing; core temperature inferred indirectly; affected by emissivity and ambient conditions
Acoustic/Ultrasonic	Mechanical waves, gas evolution	Edge/Hybrid	Feature-based ML, anomaly detection	Detects internal structural faults, early gas formation	Noise sensitive; sensor placement and coupling complexity
Electromagnetic/RF	Field perturbation, impedance proxies	Hybrid/Cloud	Deep learning, regression	Enables contactless internal state estimation and scalable monitoring	Calibration challenges; electromagnetic interference susceptibility
Impedance Proxy (Contactless EIS)	Indirect electrochemical response	Cloud/Hybrid	LSTM, Gaussian processes	High sensitivity to aging mechanisms and degradation tracking	Frequency tuning complexity; higher hardware cost
Multimodal Fusion	Thermal + Acoustic + EM	Hybrid	Attention models, deep fusion networks	High diagnostic accuracy and robustness under noise	Increased system complexity and higher data requirements

**Table 2:** Performance comparison of different contactless and wireless battery-monitoring modalities.

Modality	Target Parameter	Typical Accuracy/Error	Latency	Power Level	Main Limitation
Thermal IR	Surface temperature/thermal runaway detection	<1–3 °C	ms–s	mW	Measures surface only; core temperature must be estimated
RFID/NFC Tags	Temperature monitoring	±1–2 °C	ms–s	Passive	Signal blocked or weakened by metallic enclosures
Acoustic/Ultrasonic	Internal gas/fault detection	80–95% classification accuracy	ms–s	Low–mW	Sensitive to noise and sensor placement
RF/EM Resonance	SoC/SoH proxy	2–5% SoH RMSE	ms–s	μW–mW	Susceptible to electromagnetic interference
mmWave Radar	SoC/capacity estimation	85–95% classification accuracy	ms + model time	mW–W	Alignment and multipath reflection issues
Contactless EIS Proxy	State of Health (SoH)	1–4% SoH RMSE	Seconds	mW–W	Higher hardware complexity
Wireless Monitoring (Wired sensors + RF)	SoC/health telemetry	1–3% SoC MAE	ms–s	mW	Not fully contactless; sensors still attached

## Data Availability

No new data were created or analyzed in this study. Data sharing is not applicable to this article.

## Nomenclature

EV	Electrical Vehicles
IoT	Internet of Things
UAV	Unmanned Aerial Vehicles
LIBs	Lithium-ion Batteries
SoC	State of Charge
IR	Infrared
RFID	Radio-Frequency Identification
AE	Acoustic Emission
MCS	Micro Control Sensor
EMI	Electromagnetic Interference
MCU	Micro Control Unit
SLB	Second-Life Battery
QoS	Quality of Service
WSN	Wireless Sensor Networks
BMS	Battery Management System
WBMS	Wireless Battery Management System
EIS	Electrochemical Impedance Spectroscopy
SoH	State of Health
UHF	Ultra High Frequency
NFC	Near-Field Communication
VRBs	Vanadium Redoxflow Batteries
BackCom	Backscatter Communication
LSTM	Long Short-Term Memory
BLE	Bluetooth Low Energy
MAC	Medium Access Control
ESS	Energy Storage System
